# Influence of Oral Habits on Pediatric Malocclusion: Etiology and Preventive Approaches

**DOI:** 10.7759/cureus.72995

**Published:** 2024-11-04

**Authors:** Hattan S Katib, Amatullah A Aljashash, Abdulmajeed F Albishri, Aiysha H Alfaifi, Shatha F Alduhyaman, Mohammed M Alotaibi, Thamer S Otayf, Ranin A Bashikh, Joud A Almadani, Arwa M Thabet, Khalid A Alaman

**Affiliations:** 1 Orthodontics, King Fahd Hospital, Madinah, SAU; 2 Dentistry, Comprehensive Specialized Clinics for the Security Forces, Wadi Al Dawasir, SAU; 3 General Dentistry, King Abdulaziz Medical City Riyadh, Riyadh, SAU; 4 General Dentistry, King Khalid University, Abha, SAU; 5 General Dentistry, Dr. Abdulaziz Al Ajaji Dental Clinics, Dr. Sulaiman Al Habib Medical Group, Buraydah, SAU; 6 General Dentistry, Al Enaya General Medical Group, Taif, SAU; 7 General Dentistry, Jazan University, Jazan, SAU; 8 General Dentistry, Ministry of Health, Jeddah, SAU; 9 General Dentistry, Ministry of Health, Madinah, SAU; 10 General Dentistry, King Saud University, Riyadh, SAU

**Keywords:** malocclusion, oral habits, pediatric dentistry, thumb sucking, tongue thrusting

## Abstract

Teeth malocclusion refers to the misalignment of teeth and improper occlusion of the upper and lower jaws when the mouth is closed, which can lead to both aesthetic and functional issues such as difficulties with chewing, biting, and speech. These challenges may further contribute to broader health concerns. One of the major contributing factors to malocclusion in children is the presence of certain oral habits. Research has established a strong association between habits such as thumb sucking, tongue thrusting, and mouth breathing and various malocclusion patterns, including anterior open bite, proclination, crowding, and posterior crossbite. This review seeks to examine these three primary deleterious oral habits, their impact on malocclusion, and effective prevention strategies to address these habits in pediatric patients.

## Introduction and background

Malocclusion is the malpositioning of the upper and lower teeth that disturbs the stomatognathic complex, affecting aesthetics, function, and facial harmony [1]. A developmental condition seen globally, malocclusion is more frequent during the mixed dentition stage [2]. Studies have reported its prevalence in pediatric patients, with 79.4% of children affected by this condition, which has an impact on their psychosocial well-being [3,4].

As a complex condition, malocclusion must be considered in all three planes (sagittal, vertical, and transverse) to form an accurate diagnosis and appropriate treatment plan. Furthermore, it is important to understand the etiology of malocclusion to manage this condition effectively. Genetic and environmental influences are among the most common causes of malocclusion [1]. The developmental causes of malocclusion include disturbances during embryonic development, growth disturbances in the fetal and perinatal periods, progressive deformities in children, disturbances arising in adolescence or early adulthood, and dental developmental disorders [5]. One major environmental factor is the prevalence of harmful oral habits, such as mouth breathing, thumb sucking, and tongue thrusting [5]. However, recent literature has described these notorious habits (e.g., mouth breathing) as risk factors rather than etiological factors [6].

While children are still in their growth phase with moldable bony structures, physiological habits such as chewing, speaking, and normal swallowing play a vital role in jaw growth. In contrast, if deleterious habits persist beyond the age of three to four years and after the emergence of permanent dentition, they tend to disrupt the balance between dental structures, oral musculature, and occlusal function [7]. Since infants and young children unconsciously engage in these oral habits as part of their reflexes, feelings of discomfort or fear may encourage these habits and therefore negatively affect their growth and development [1]. Moreover, Garde et al. and Perillo et al. described mouth breathing, lip sucking and biting, nail biting, and digit sucking as repetitive behaviors that also harm the quality of life, as reported by most pediatricians [7,8]. However, the degree of impact varies according to the nature, onset, and duration of these habits [7]. Furthermore, the patient’s age and gender are also important factors to consider.

By understanding the etiology of malocclusion, pediatric dentists will be able to focus on addressing the root cause. Therefore, the objective of pediatric dentistry is to guide dental development from early childhood to achieve well-aligned occlusion in the long term [1]. The key to correcting malocclusion is to eliminate unwanted oral habits and intervene at an early stage (pre-adolescence and adolescence) to minimize their effects [7].

This review aims to identify the association between deleterious oral habits, such as mouth breathing, tongue thrusting, and thumb or digit sucking, with the development or progression of malocclusion in pediatric patients. Additionally, the review seeks to recognize abnormal oral habits in children from four to 18 years old, enabling timely intervention and better outcomes in the future. This review will help dental practitioners and pediatric dentists understand the science behind different oral habits and explore treatment approaches that will improve outcomes for pediatric patients.

## Review

Methodology

A manual search was carried out on databases such as PubMed, Google Scholar, Cureus, and ScienceDirect. The following keywords were used: “oral habits”, “malocclusion”, “pediatric patient”, “mouth breathing”, “tongue thrusting”, and “thumb sucking”. All relevant literature, including cross-sectional studies, narrative reviews, and surveys, was reviewed. Only studies published in English and focused on pediatric patients were included. Studies discussing multiple oral habits, which provided larger data from specific regions of the world, were also considered. Based on their titles, studies advocating malocclusion in adult patients were excluded. Additionally, repetitive research studies were excluded.

The primary objective of this review was to elucidate the correlation between deleterious oral habits and malocclusion in pediatric patients. Secondly, the review aimed to understand methods for early interception of these habits and ways to prevent malocclusion. Both recent and seminal studies were included to comprehensively achieve these objectives.

Discussion

Malocclusion becomes visible in the dentition when permanent teeth begin to erupt. Therefore, pediatric patients with late mixed and early permanent dentition are more reliable sources of statistics regarding the association between lingering oral habits and dental misalignment [9]. Accordingly, studies have confirmed a higher prevalence of malocclusion in pediatric patients who have deleterious oral habits [6,7].

Zakirulla et al. identified mouth breathing, thumb sucking, and tongue thrusting as significant etiologic factors leading to anterior teeth crowding, open bite, proclination, and spacing. They emphasized the need for health education, awareness, preventive strategies, and early intervention in pediatric patients to cease these deleterious oral habits [9]. An interesting phenomenon discussed in the current body of research is the prevalence of oral habits according to the age and gender of the patient. In their survey, Melo and Pontes found that the incidence of nail biting and mouth breathing was more common in girls, while bottle-feeding was a frequent occurrence in boys. Additionally, they found that between the ages of three and five years, mouth breathing, bottle use, bruxism, and object biting were more prevalent [10]. Further, a questionnaire-based study was conducted among six- to 12-year-old school-going children; the researchers found that tongue thrusting was common among children aged eight to 10 years and bottle-feeding among children aged six to eight years. Additionally, girls engaged more in deleterious oral habits than boys [7].

With a growing research interest in diagnosis and treatment planning for malocclusion, it is equally important to delve into the prevalence of deleterious oral habits, the associated malocclusion, and methods to prevent them in pediatric patients [11]. Understanding the causative factors of malocclusion is important for successful orthodontic treatment, and initial correction always requires cessation of poor oral habits. At the early stages of malocclusion, preventative treatment should receive more attention than orthodontic treatment to avoid further complications and treatment-related complexities in the future [5].

Types of deleterious oral habits

Sucking and other habits are considered normal up until the primary dentition years, as the changes are reversible. However, if these habits persist beyond that age into the permanent dentition years, they become problematic. This review explores the three main deleterious oral habits, their association with malocclusion, and how to prevent them in pediatric patients.

Mouth Breathing

Humans are originally nasal breathers. However, the alteration of the respiratory pattern from nasal to oral is a key contributor to malocclusion, as it influences the jaw, tongue, and, to a lesser extent, head posture. This shift can be primarily due to partial nasal obstruction caused by adenoids or enlarged pharyngeal tonsils through allergic rhinitis, which eventually becomes a habit even after the removal of the obstruction, or, in rare cases, due to total nasal obstruction [5]. Symptoms can include a lack of nasal airflow, sneezing, itching, snoring, obstructive sleep apnea, and respiratory infections [12]. Due to enlarged adenoids, the term associated with this type of malocclusion is “adenoid facies,” characterized by a narrow arch, proclined teeth, and lips separated at rest [5].

In contrast, one study’s population disregarded the obstructive size of adenoids or tonsils and the presence of rhinitis as risk factors for developing malocclusions, such as Angle’s Class II malocclusion, anterior open bite, and posterior crossbite [13]. In their review exploring the association between oral habits and malocclusion, Kharat et al. thoroughly explained that mouth breathing, aside from being a habitual activity, can also occur due to prolonged inflammation of the nasal mucosa associated with allergies or chronic infections [14]. Additionally, research on mouth-breathing habits in pediatric patients found that different inter-arch relationships can be observed in individuals due to varying facial genotypes [14].

Digit Sucking Habits

Digit sucking habits is categorized as non-nutritive sucking habit. Children engage in this habit more frequently because it is a comforting maneuver, and they may develop a strong sense of emotional attachment to it [9,14,15]. Within this category, pacifier use and digit sucking are also studied [16]. The habit typically begins as a primitive reflex observed in infants. Some studies have associated non-nutritive sucking with situations related to fatigue, boredom, fear, excitement, hunger, and both physical and mental distress. This behavior provides comfort and a sense of security during stressful periods [14]. Ferrante and Ferrante reported that thumb sucking stimulates nasopalatine receptors, creating a muscular balance that relieves both mental and physical stress [17].

Active vs. Passive Digit Sucking Habits

The classification of thumb sucking into active and passive habits highlights the varying degrees of intensity and impact on dento-skeletal development. An active habit involves forceful sucking with noticeable pressure applied by the thumb or finger against the teeth and surrounding tissues. This intense habit typically exerts prolonged forces that can result in significant dental malocclusions, such as anterior open bite, maxillary protrusion, and posterior crossbite. In contrast, a passive habit involves minimal or no force, where the digit is placed in the mouth without continuous sucking pressure. While passive sucking may cause some displacement of teeth if it persists beyond the normal age, its impact is usually less severe compared to active habits [5,14,15].

Impact on Malocclusion and Orthodontic Considerations

The core reason for malocclusion due to digit sucking habits lies in the direct pressure on teeth and the alteration of cheek and lip resting patterns [18]. According to the equilibrium theory, the severity of malocclusion depends more on the duration of the habit - the number of hours per day the child sucks their thumb - rather than the magnitude of the force applied [5,14]. Thumb sucking is generally considered normal until the age of three to four years, and if it ceases within this time frame, cheek and lip pressures often restore the teeth to their natural alignment [5,14]. However, more recent research has classified thumb and lip sucking as complex neuromuscular patterns that should be addressed if they persist beyond three years of age [2]. Similarly, Nasir and Nasir indicated that non-nutritive sucking habits typically cease spontaneously between two and four years [19]. If the habit continues into later childhood, orthodontic intervention may become necessary, especially as permanent teeth begin to erupt [5].

Persistent digit sucking habits beyond the normal age can interfere with dento-skeletal development, leading to more severe malocclusions [1,14]. Prolonged habits may necessitate early orthodontic treatment using appliances such as thumb cribs or palatal guards to discourage the behavior. Additionally, visible signs such as bite marks or deformation of the thumb can indicate the continuation of the habit [2].

Tongue Thrusting

The physiological swallowing pattern of an adult involves positioning the tip of the tongue at the level of the incisive papilla when the dental arches are in contact. As a result, the contraction of perioral muscles is minimal, the teeth are in contact for a shorter period, and there is neither tongue thrust nor forward posture during deglutition [14]. Any deviation from this swallowing pattern is known as atypical swallowing. Atypical swallowing is a common occurrence in pediatric patients and rarely occurs in isolation; it is most often associated with prolonged thumb sucking and mouth breathing. Tongue thrusting is a condition during atypical swallowing where the tongue makes contact with any teeth anterior to the molars while swallowing [20]. This abnormal positioning of the tongue may lead to both dental and skeletal anomalies.

The tongue plays a major role in respiration, mastication, speaking, and swallowing. In a normal swallowing pattern, there is no tongue thrust since the tongue rests on the lingual surface of the maxillary anterior dentoalveolar area. Tongue thrust swallowing is a normal pattern in infants, but by the age of two to four years, a functionally mature swallowing pattern emerges [21]. The prevalence of this habit among young children between the ages of four and six years has been reported in various studies as ranging from 40% to ⁠80% [22,23], while for children aged 12-15 years, the prevalence is reported as 3-⁠25% [23,24].

Mechanisms behind how deleterious oral habits lead to malocclusion

Mouth Breathing

Mouth breathing due to airway obstruction involves lowering the mandible and tongue and tipping the back of the head. Over a long period, this continued posture can lead to an increase in anterior facial height, lip incompetence, and supra-eruption of posterior teeth. The mandible rotates down and back, resulting in the opening of the bite anteriorly and an increase in overjet. In addition, excessive pressure from the stretched cheeks can cause the narrowing of the maxillary dental arch [5]. Kharat et al. also mentioned the occurrence of anterior open bite and a narrow arch, with or without posterior crossbite [14]. They further depicted the incidence of Class II malocclusion in such patients. Holding the open-mouthed posture for an extended duration, whether due to habit or enlarged tonsils, leads to reduced transverse maxillary growth and results in a posterior crossbite [14]. Souki et al., in their cross-sectional descriptive study, also found a positive relationship between mouth breathing and the occurrence of posterior crossbite [13]. However, further studies are needed to establish a definitive relationship between mouth breathing and posterior crossbite [14].

Existing literature has confirmed the influence of mouth breathing on malocclusion. Grippaudo et al. found that mouth breathing plays a role in the etiopathogenesis of tooth malposition and the alteration of skeletal growth. It was highlighted that in such patients, there is a greater risk of malocclusion due to a higher genetic predisposition and an unfavorable growth pattern [12]. Several studies on the influence of mouth breathing on oral health share a consensus that the habitual insufficiency of nasal breathing alters the pattern of craniofacial growth due to unstable muscle functionality [25,26]. Therefore, early recognition of the habit or the associated underlying condition is recommended for timely intervention.

Digit Sucking Habits

Depending on duration, frequency, and intensity, thumb sucking may contribute to both thumb deformities and dental occlusion. The abnormality of dental occlusion is often accompanied by disruptions in dental arch characteristics [14]. The relationship between thumb sucking and dental disturbances has been widely studied. Non-nutritive sucking habits are mainly associated with anterior open bite, excessive overjet, and Class II canine and molar relationships [14]. Further studies have elaborated on the mechanism by which thumb sucking leads to disturbances in the dental apparatus. Thumb sucking displaces the tongue to a lower position, which alters the balance between the outward thrust of the tongue and the inward contraction of the cheek muscles, resulting in a narrow maxillary arch [1]. Additionally, due to excessive pressure on the thumb between the anterior teeth, the lower incisors are pushed lingually, and the upper incisors are displaced labially. Consequently, various complications such as anterior open bite, protrusion of the premaxilla, atypical swallowing, and posterior crossbite may develop [1].

The anterior open bite is caused by the interruption of the normal eruption of incisors and the supra-eruption of posterior teeth. Proffit et al. explained that 1 mm of over-eruption of posterior teeth results in 2 mm of anterior bite opening. Furthermore, the downward positioning of the mandible allows accommodation of the thumb between the anterior teeth [5]. The V-shaped maxillary arch results from increased cheek pressure at the corners of the mouth. The narrowing of the upper arch leads to the development of crossbite, which requires close attention from orthodontists, as it will not correct itself spontaneously. In contrast, the digit sucking habit guides the mandible forward, potentially leading to an edge-to-edge bite or anterior crossbite [1]. It can also cause an asymmetrical open bite, which is typically worse on the side where the digit is sucked. However, not all digit suckers will develop an anterior open bite, as its occurrence depends on the duration and frequency of the habit. Children who suck for more than six hours a day often develop significant malalignments [14].

The literature consistently agrees on the direct link between thumb or digit sucking and various malocclusions in pediatric patients. It is important to recognize that certain malocclusions will require orthodontic therapy, and cessation of the thumb sucking habit should be encouraged before any treatment plan is implemented [5].

Tongue Thrusting

Kharat et al. proposed a possible connection between tongue thrusting and the development of malocclusion. When the tongue is postured forward and rests between the incisors, it may obstruct incisor eruption and lead to an anterior open bite. The diagnostic feature for this, seen on a lateral cephalogram, will show a reverse curve of Spee in the lower arch due to the infra-eruption of the incisors. Clinically, orthodontists can easily differentiate between a mature swallower and a tongue-thrust swallower [14]. Not only does the tongue move forward, but other common signs of tongue thrusting include contraction of the perioral muscles, hyperactivity of the buccinators, and swallowing without brief tooth contact. Additionally, tongue thrusting may affect oral sensory perception, impairing motor activity and further aggravating malocclusion [21].

Although the literature has shown a correlation between tongue thrust and the occurrence of dental malocclusion in pediatric patients, there remains a need for further studies with larger population sizes. Questionnaire-based research should also be performed to better elucidate the process of tongue thrusting and its influence on malocclusion.

Consequences of Untreated Oral Habits

The present literature shows a strong correlation between untreated and prolonged deleterious oral habits and malocclusion. Having understood the pathophysiology of these habits and their prevalence in pediatric patients, it is important to discuss the emergence of major and minor alterations attributed to uncontrolled habits. This information will allow clinicians and parents to foster healthy oral habits in children from a very young age, thereby avoiding further oral health-related problems.

Rakosi and Schilli established the role of mouth breathing in the development of some forms of Class III malocclusion with reduced or reverse overjet. The children in their study clinically presented with open mouths, low-postured tongues, and excessive mandibular growth. Additionally, the lack of tongue thrust on the palate and upper jaw may lead to sagittal and transverse maxillary deficiencies, confirming the development of Class III malocclusion [27].

In their cross-sectional study on 3,017 children using the ROMA index, Grippaudo et al. found that mouth breathers had narrow dental arches that left the upper arch profoundly crowded. Additionally, abnormal lip function was observed, with the lower lip being large and bulbous and the upper lip short and functionless. They concluded that mouth breathing leads to increased overjet, open bite, anterior or posterior crossbite, and displaced contact points [12]. Harari et al. and Stokes and Della Mattia suggested the typical features of mouth breathers include a long face, dark circles, narrow nostrils, a narrow upper arch, a high-arched palate, and a gummy smile. It is also associated with Class II or Class III malocclusion with a higher prevalence of posterior crossbite and anterior open bite [28,29].

The study by Garde et al. indicated that long-term non-nutritive sucking (thumb or digit sucking) creates various problems due to disturbances between external and internal muscle forces [7]. These habits are considered normal in infants and young children and are usually associated with their need for contact and security [30]. However, excessive and continued indulgence beyond the age of three years can lead to poor dental health and speech clarity issues. Thumb sucking and nail biting habits can also lead to infectious diseases, as it was found that subjects with these oral habits had higher levels of *Escherichia coli* and Enterobacteria compared to children without such habits [7].

Tongue thrusting is commonly associated with anterior open bite, abnormal speech, and the proclination of maxillary anterior teeth [7]. Dixit and Shetty performed a comparative analysis of soft tissue, dental, and skeletal morphologic characteristics in children with and without tongue-thrusting habits [21]. They selected 42 children aged 10-14 years, comprising 21 children with tongue thrust and 21 without. The children with tongue thrust were noted to experience lip incompetency, open bite, parafunctional maxillary incisors, and lisping, and they were more inclined toward mouth breathing. There was no difference in the angulation of mandibular incisors, inter-molar, or inter-premolar width, and the skeletal parameters remained unchanged [21]. Children with atypical swallowing exhibit contraction of the mentalis muscle, interposition of the lower lip between the dental arches, and mutual movements of the head and neck. This can also lead to temporomandibular disorders and alterations of the facial profile, with a hypertonic chin and hypotonic orbicularis oris muscle [20].

Several studies have confirmed the occurrence of both short-term and long-term changes due to deleterious oral habits in children. Short-term changes may be reversible after the discontinuation of the habit and may not require treatment. However, long-term changes can result in serious dental issues that require prompt intervention and management. One such long-term complexity of deleterious oral habits is Angle’s malocclusion. Angle’s malocclusion necessitates comprehensive orthodontic treatment, as these habits interfere with the growth and development of the stomatognathic system and orofacial musculature, leading to changes in teeth alignment. Ferreira et al. studied 140 patients to verify the presence of deleterious oral habits in pediatric patients with malocclusion and observed the incidence of Angle’s malocclusion in subjects aged six to 10 years and 11 months [31]. They found that 67.1% of the subjects had a history of deleterious oral habits, with a predominance of Class II malocclusion, followed by Class III and Class I. The increased prevalence of Class II malocclusion pointed toward Class II Division 1, as these patients presented with proclined incisors and increased overjet [31]. Rodríguez-Olivos et al. discovered that children with atypical swallowing habits were more likely to develop malocclusion in all three planes of space [2]. Among 155 evaluated patients, 45.3% had vertical malocclusion, 52% had sagittal malocclusion, and 13.6% had transverse malocclusion. In the sagittal plane, Class III malocclusion was more common in males, while Class II Division 1 was more common in females [2].

Finally, the persistence of such habits and the development of malocclusion in pediatric patients also impact psychosocial well-being and quality of life. Carminatti et al. [32] concluded that deleterious oral habits, such as pacifier sucking and mouth breathing, negatively affect the quality of life in children [32]. A cross-sectional study on 451 preschool children aged three to five years found that children with anterior open bite associated with deleterious oral habits experienced a decline in quality of life [33]. An anterior open bite poses new challenges and functional disabilities for the child, such as difficulty eating and pronouncing words, which can carry into later stages of dentition if left untreated [34].

The close link between dental changes and harmful oral habits has been well established in this review. The emergence of malocclusion in all three planes, changes in soft tissue anatomy, susceptibility to various infectious diseases, and a negative impact on a child’s lifestyle, affecting their self-confidence, were the most common outcomes in pediatric patients with bad oral habits [5]. Therefore, a multidisciplinary approach and early intervention are important in addressing these harmful etiological factors to prevent malocclusion and future problems. If malocclusion has already developed, an early orthodontic approach should be initiated to promote favorable growth and ideal tooth positioning (Table 1).

**Table 1:** Malocclusion in all three planes and associated habits

Planes of malocclusion	Type of malocclusion	Associated oral habits
Sagittal	Excessive overjet [5]	Thumb sucking and mouth breathing [5]
Sagittal	Class III malocclusion [27]	Mouth breathing and lack of tongue thrust [27-29]
Sagittal	Class II malocclusion [28,29,31]	Thumb sucking [31] and mouth breathing [28,29]
Vertical	Anterior open bite [1,5,14,21]	Thumb sucking [1,14], tongue thrusting [7,21], and mouth breathing [5]
Transverse	Posterior crossbite [13,14]	Mouth breathing [14], and thumb sucking [13]

Preventive and interventional approaches

The extant literature primarily focuses on the early cessation of these oral habits and the incorporation of preventive approaches at a younger age so that malocclusion can be avoided [7,35]. The first approach to prevent these oral habits from causing malocclusion is to control and stop these reflex activities [2]. There are various methods to break the habit. Fernandes and Lima suggested that parents and teachers of school-going children should be vigilant in noticing such harmful habits early so that different strategies can be applied, especially in the school environment, to control the habit and avoid the overuse of pacifiers and baby bottles [36]. Preventive measures should be initiated early so that deleterious oral habits do not persist for long. Parents should be advised to breastfeed for a minimum of six months, replace finger sucking with an orthodontic pacifier, and limit bottle use to only until the age of three [5].

Silva et al. reinforced the idea already established in this review that there is a strong correlation between oral habits, such as mouth breathing and atypical swallowing, and malocclusion across different planes of space. They added that atypical swallowing habits must also be diagnosed early and carefully managed [35]. Oral breathing is widely accepted in the literature as a cause of dental malocclusion. Since mouth breathing is associated with several systemic and developmental disorders and is known to exacerbate preexisting malocclusion, early intervention is recommended [20]. Early diagnosis of underlying causes, such as respiratory obstruction, can help prevent other craniofacial abnormalities. The management of a patient with mouth-breathing habits should follow a systematic approach. First, consideration of the child’s age is important. All infants should be tested for craniofacial deformities that may compel the child to shift to oral breathing. Moreover, oral breathing is often self-corrected after puberty when nasal obstructions are reduced as the child’s craniofacial bones, dental occlusions, and facial soft tissues develop [37]. Second, an evaluation by an otorhinolaryngologist (ENT) is essential to determine whether the problem lies in the tonsils, adenoids, or nasal septum. In habitual cases, the condition may persist even after removal of the cause [37]. Finally, prevention and interception of the habit through early oral screening are crucial to managing the issue effectively [37]. The impact of deleterious oral habits and developing malocclusion should be managed through a multidisciplinary approach involving pediatricians, dentists, and psychologists. For instance, controlling the impact of mouth-breathing habits requires collaboration involving a pediatrician, allergist, ENT specialist, speech therapist, and orthodontist. Early detection of underlying causes, such as allergic rhinitis or adenotonsillar hypertrophy, allows for timely treatment, preventing further worsening of the already-developed malocclusion [12]. Additionally, early orthodontic intervention in young patients can yield more stable results, with less extraction of permanent teeth, shorter treatment durations, and reduced risk of enamel demineralization and periodontal diseases post-treatment [38].

Digit sucking habits can be managed through various strategies, including parental advice, behavioral therapy, and orthodontic interventions. Behavioral modification techniques, such as positive reinforcement, reward calendars, counseling, and applying bitter-tasting substances to the thumb or finger, are commonly used to discourage the habit [16,39,40]. Myofunctional therapy, which includes exercises aimed at reeducating the muscles involved in swallowing, speech, and resting tongue posture, is another noninvasive approach that may be effective [41-47].

Managing Active vs. Passive Digit Sucking Habits

The management of digit sucking habits varies depending on whether the habit is active or passive, and this distinction is critical in tailoring treatment. Active digit sucking involves forceful and frequent sucking, applying significant pressure to teeth, alveolar bone, and surrounding soft tissues [47]. This habit often results in more severe dento-skeletal issues, such as anterior open bites, posterior crossbites, or maxillary protrusions, which can worsen over time [1,14]. Behavioral strategies alone may not suffice for active habits, requiring early orthodontic intervention with devices such as palatal cribs, cage-type appliances, or spurs to prevent malocclusion [15,40]. These habits are also associated with a higher likelihood of relapse, even after initial treatment, necessitating long-term follow-up [44]. Passive digit sucking, on the other hand, involves minimal force, with the thumb or finger remaining in the mouth without active sucking pressure. While passive habits generally cause less severe malocclusion, they may persist unnoticed for longer periods, as the dental impact is subtler [47]. Behavioral interventions, including positive reinforcement, counseling, and reward calendars, are more likely to be effective for passive habits. Regular follow-up is essential to monitor progress and ensure the habit does not evolve into an active form. If passive digit sucking habits persist beyond age four despite behavioral strategies, referral to a pediatric dentist is recommended [15,19].

Krishnappa et al. reported a novel behavioral management strategy that involved an alarm-clock-activated wristwatch designed to alert an eight-year-old child whenever digit sucking began. This device successfully stopped the habit within five months, with continued use of the watch for six months to prevent relapse [40]. Customizing treatment strategies to suit the specific type of habit, whether active or passive, is crucial for long-term success.

Long-Term Management and Prognosis

Nonsurgical and non-orthodontic treatments for anterior open bites caused by digit sucking habits include myofunctional therapy and Stomahesive wafers, which guide the tongue to rest on the incisive papilla [41]. Regular oral evaluations are essential during the transitional phase (ages three to six), when permanent teeth begin to erupt, as this period is critical for identifying habits that might affect permanent dentition [39]. If digit sucking habits cease during the mixed dentition stage, many dental changes reverse naturally. However, appliance therapy may be needed for cases with moderate malocclusion risk [42].

Sustained motivation from children, parents, and caregivers is essential for effective treatment, particularly for active habits that carry a higher risk of relapse [42]. The prognosis for digit sucking-induced malocclusion is generally favorable, as mild skeletal changes often self-correct once the habit stops [43]. However, in severe cases, orthognathic surgery may be necessary, and there remains a risk of relapse if treatment is not comprehensive [44].

Tongue thrusting is a normal phenomenon in neonates, where the tongue is positioned between the gum pads and the mandible is stabilized by facial muscles during swallowing. This behavior lasts until primary dentition erupts. During normal mature swallowing, the tongue rests high on the palate behind the maxillary incisors, with no lip or cheek activity during swallowing. In the mixed dentition stage, transitional swallowing patterns are typically self-correcting. In cases of thumb sucking and the resulting open bite, the tongue thrusts forward to achieve a lip seal, creating a simple tongue thrust. Complex tongue thrusts are seen in subjects with diffused open bites such as mouth breathers. Retained infantile swallowing occurs when this behavior persists beyond the eruption of permanent teeth. Pediatric dentists should intervene at all stages - simple and complex tongue thrusts and retained infantile swallows [45].

Proffit and Mason studied treatment methodologies for pediatric patients with tongue thrusting habits and recommended myofunctional therapy only when speech or dental issues arise. If tongue thrusting persists beyond puberty, tongue therapy combined with orthodontic treatment is indicated. Speech therapy may also be necessary to address speech errors and reposition the tongue tip posteriorly [46]. Shah et al. suggested using myofunctional appliances as a form of active treatment for tongue thrust, along with habit-breaking appliances like tongue cribs to restrict anterior tongue movement [45]. The ideal age to begin myofunctional therapy remains debated, with some recommending it before age 10 and others suggesting starting at age 10 or later. Children younger than five do not benefit much from speech therapy, but success is seen in children aged five to 13 [20]. As a form of passive treatment, orthodontic intervention is indicated when necessary. However, controlling the habit is essential, and only after its elimination should corrective appliances be used to prevent relapse [45].

Certain malocclusion cases can be resolved with orthodontic therapy. To ensure effective treatment, it is important to understand the timing of craniofacial growth, especially since the vertical component is the last to end. Failure to manage this aspect can lead to relapse [20]. Thus, successful treatment outcomes depend on cooperation between the child and the dentist, early intervention, effective approaches to eliminate oral habits, motivation to prevent relapse, and a multidisciplinary approach.

Clinical recommendations and future directions

In pediatric patients, oral examinations are an essential component of assessing for potential complications arising from deleterious oral habits. Early screening and regular dental checkups ensure early detection. The first examination should begin when the first deciduous tooth erupts in the oral cavity, at around six months of age. Additionally, tracking oral habits using smart devices and apps that provide real-time feedback could enable early intervention and increase patient compliance. Healthcare professionals should be aware of all the harmful oral practices in a child and their negative effects on dental soft and hard tissues. When these habits persist beyond the acceptable age, referral to a specialist or orthodontist becomes mandatory [20]. A multidisciplinary approach involving pediatricians, speech therapists, and orthodontists should then be employed. While pediatricians hold expertise in child health, it is equally important to consult designated specialists to ensure desirable long-term outcomes for the child [5].

Although the availability of extensive literature has made it easier to assess, diagnose, and design methodologies to control or prevent the issue, there are still gaps in the research that need to be addressed. Prospective studies should focus on conducting long-term research on the progression of malocclusion related to deleterious oral habits so that stepwise management can be initiated for optimal outcomes. There is also a need to develop innovative habit cessation strategies, such as digital behavioral therapy, biofeedback mechanisms, and novel screening tools, to identify children who are at risk and intervene before severe issues arise. Lastly, further studies are needed to explore how genetic influences interact with environmental factors, such as oral habits, to affect the severity and type of malocclusion.

## Conclusions

This review provides dental practitioners and pediatric dentists with a deeper understanding of the science behind various oral habits, how they lead to dental and skeletal malocclusions, and the treatment approaches that can shift the equilibrium in favor of the child patient. Genetic and environmental influences have been identified as the most common causes of malocclusion, with deleterious oral habits such as mouth breathing, thumb sucking, and tongue thrusting acting as key environmental factors. These habits are adaptive and normal up to a certain age, typically disappearing spontaneously. However, when these habits persist beyond the age of three to four years and after the emergence of permanent dentition, they disrupt the harmony of the dental structure, oral musculature, and occlusal function.

If left unaddressed, mouth breathing, thumb sucking, and tongue thrusting can lead to a variety of malocclusions. Some of these may become severe to the point where orthognathic surgery and orthodontic treatment are required. A multidisciplinary approach involving pediatric dentists, speech therapists, school teachers, and parents is essential to intercept these habits and prevent the development of malocclusion. The child’s motivation to give up the habit is a crucial factor, and all efforts should be focused on stopping the habit to enhance the effectiveness of the treatment. Early intervention and preventive strategies not only minimize harm but also help the child regain self-confidence and improve their quality of life. Further research is needed to explore early screening methods and effective preventive approaches. Additionally, long-term studies are needed to better understand the influence of genetic predisposition on malocclusion related to oral habits.
